# *TaRar1* Is Involved in Wheat Defense against Stripe Rust Pathogen Mediated by *YrSu*

**DOI:** 10.3389/fpls.2017.00156

**Published:** 2017-02-14

**Authors:** Xiaojing Wang, Yaru Wang, Peng Liu, Yan Ding, Xiaoqian Mu, Xiping Liu, Xiaojie Wang, Mengxin Zhao, Baoyu Huai, Li Huang, Zhensheng Kang

**Affiliations:** ^1^State Key Laboratory of Crop Stress Biology for Arid Areas, College of Life Sciences, Northwest A&F UniversityYangling, China; ^2^State Key Laboratory of Crop Stress Biology for Arid Areas, College of Plant Protection, Northwest A&F UniversityYangling, China; ^3^Department of Plant Sciences and Plant Pathology, Montana State University, BozemanMT, USA; ^4^China–Australia Joint Research Centre for Abiotic and Biotic Stress Management, Northwest A&F UniversityYangling, China

**Keywords:** *TaRar1*, *Puccinia striiformis* f. sp. *tritici*, salicylic acid, reactive oxygen species, virus-induced gene silencing

## Abstract

RAR1 is a eukaryotic zinc-binding protein first identified as required for race-specific resistance to powdery mildew in barley. To study the function of TaRAR1 involvement in wheat (*Triticum aestivum* L.) defense against the infection of stripe rust pathogen *Puccinia striiformis* f. sp. *tritici* (*Pst*), we identified and cloned three wheat homeologous genes highly similar to the barley *HvRar1*, designated as *TaRar1-2A, TaRar1-2B*, and *TaRar1-2D*. The three TaRAR1 proteins all contain two conserved cysteine-and histidine-rich domains (CHORD-I and -II) shared by known RAR1-like proteins. Characterization of *TaRar1* expression revealed that the expression was tissue-specific and up-regulated in wheat during stripe rust infection. Moreover, the transcription of *TaRar1* was induced by methyl jasmonate, ethylene, and abscisic acid hormones. The same results were observed with drought and wound treatments. After *TaRar1* was silenced in wheat cultivar Suwon11 containing the stripe rust resistance gene *YrSu*, the endogenous salicylic acid (SA) level, the hydrogen peroxide (H_2_O_2_) accumulation and the degree of hypersensitive response (HR) were significantly decreased, and the resistance to the avirulent pathotype of stripe rust was compromised. Meanwhile, the expression of catalase, an enzyme required for H_2_O_2_-scavenging, was up-regulated. Taken together, we concluded that *TaRar1* is involved in wheat defense against stripe rust mediated by *YrSu*, and the defense was through SA to influence reactive oxygen species accumulation and HR.

## Introduction

Plants have evolved sophisticated and effective mechanisms against most potential pathogens, such as non-host resistance, race specific and race non-specific resistance (Nürnberger and Lipka, 2005; Jones and Dangl, 2006). Non-host resistance is also called species-level resistance; it means that all genotypes of a given pathogen species can’t infect all genotypes of a plant species. Race-specific resistance is also known as gene-for-gene resistance (Flor, 1971), in which plant *R* genes can recognize cognate avirulence (*Avr*) genes from the pathogens to trigger defense responses. In most cases, *R-*gene-mediated resistance triggers a complex of signal transduction cascade leading to a local programmed cell death (PCD) namely the hypersensitive response (HR), and a systemic acquired resistance (SAR) (Heath, 2000; Durrant and Dong, 2004). HR occurs at the infection sites and immediate surrounding areas to restrict the pathogen growth. SAR is often induced along with the elevated pathogenesis-related (*PR*) gene expression through salicylic acid (SA) or jasmonic acid (JA) mediated signaling pathway to generate a global and a broad-spectrum resistance in plants (Durrant and Dong, 2004).

To date, the largest class of resistance genes cloned encodes proteins containing a nucleotide binding (NB) site and leucine-rich repeat (LRR) domains. In addition to *R* genes, many genetic components are also required in the regulation of *R*-gene-mediated defense signaling (Century et al., 1995; Parker et al., 1996; Shirasu et al., 1999; Austin et al., 2002). Among them, the gene *Required for Mla12 Resistance1* in barley (*HvRar1*) was first identified as necessary for the function of multiple powdery mildew *R* genes (Jørgensen, 1994; Shirasu et al., 1999). *Rar1* was also well documented to be required in *R*-gene-specific resistance in *Arabidopsis* and *Nicotiana benthamiana* (Liu et al., 2002; Tornero et al., 2002). In wheat, *Rar1* was first reported to be involved in the *Lr21*-mediated resistance against leaf rust (Scofield et al., 2005), but was not required by the *Sr33*-mediated signaling pathway (Periyannan et al., 2013), suggesting *Rar1* is not required by every *R* gene.

Barley *Rar1* encodes a protein with two zinc-binding domains named as CHORD-I and -II (cysteine-and histidine-rich domain) (Azevedo et al., 2002). During initial yeast two-hybrid screening, an ubiquitin ligase protein containing a Skp1-cullin-F box, named SGT1, was identified as a RAR1-interacting partner (Shang et al., 2006; Tai, 2008; Cantu et al., 2013). *Rar1* and *Sgt1* are required by many but not all NB-LRR *R* genes to mediate resistances against viral, bacterial, oomycete, or fungal pathogens (Kitagawa et al., 1999; Austin et al., 2002; Liu et al., 2002; Tornero et al., 2002; Scofield et al., 2005; Periyannan et al., 2013). The molecular chaperone, RAR1 associated with SGT1 and HSP90, has been shown to regulate the correctly folded of R protein complexes and active the downstream signaling pathways (Shirasu and Schulze-Lefert, 2003).

Stripe rust disease is caused by *Puccinia striiformis* f. sp. *tritici* (*Pst*), which is one of the most common and destructive diseases of wheat (*Triticum aestivum* L.) in the world (Chen, 2005). To determine molecular mechanisms involved in wheat–*Pst* interaction, we isolated a gene highly upregulated from wheat cultivar Suwon11 infected with stripe rust fungus. The gene shares a high similarity with the barley *HvRar1* gene and the wheat *TaRAR1-1* gene, designated as *TaRar1*. The transcript abundance of *TaRar1* was studied in Suwon11 seedlings inoculated with two different *Pst* pathotypes. Additionally, the expression patterns of *TaRar1* were studied under different stresses and hormone treatments. Furthermore, the involvement of *TaRar1* in defense against *Pst* was investigated using barley stripe mosaic virus induced gene silencing. The relationships between the *TaRar1* silencing and the levels of SA accumulation and reactive oxygen species (ROS) accumulation were assayed; HR and pathogen growth were also studied. Our studies suggested that *TaRar1* plays an important role in wheat defense against *Pst* pathogen through SA to modulate ROS accumulation and HR.

## Results

### Sequence Analyses of *TaRar1* cDNA and Protein

**FIGURE 1:**
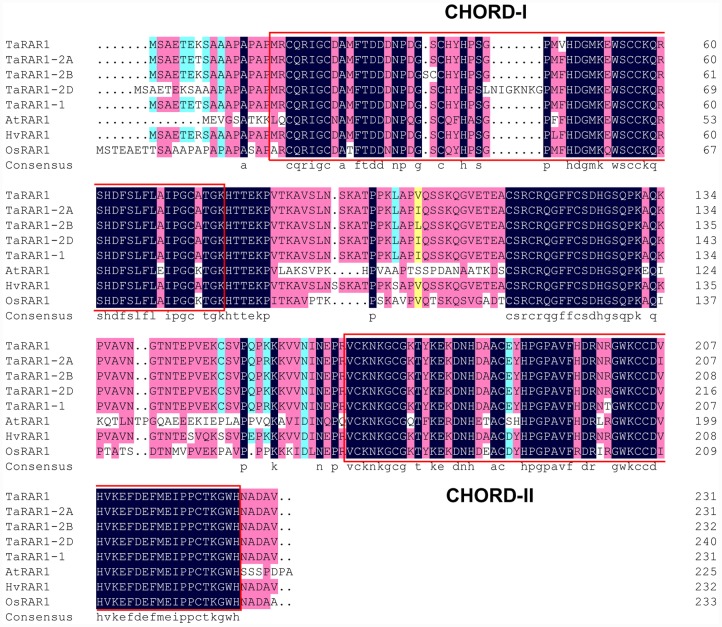
**Alignment of RAR1 proteins from different species.** Comparison of TaRAR1(KX852426), wheat alleles TaRAR1-2A, TaRAR1-2B, TaRAR1-2D, and the RAR1 protein(EF202841.1) from wheat cultivar WGRC7 with RAR1 proteins from rice OsRAR1 (XP_015623436.1), barley HvRAR1(AF192261_1) and *Arabidopsis* AtRAR1(BAB11239.1) revealed highly conserved sequences in the two conserved CHORD-I and -II domains indicated in two red boxes.

A 675-bp cDNA fragment was isolated due to its high expression level from the transcriptomes of wheat cultivar Suwon11 infected by avirulent *Pst* CYR23 at 1 day post inoculation (dpi). The cDNA shares a high homology with the *Rar1* gene of barley *HvRar1* (AF192261_1) and the wheat *TaRAR1-1* or *wRar1* amplified from WGRC7 (EF202841.1) (Tai, 2008; Cantu et al., 2013). The cDNA sequence was deposited in NCBI GenBank as *TaRar1* (KX852426). A longer 696-bp cDNA sequence was amplified from Suwon11 mRNA using primers TaRar1-F/R (**Table 1**) designed based on *TaRAR1-1*. The deduced protein has 231 amino acids, with a predicted molecular weight of 25.29 kD and an isoelectric point (pI value) of 8.11. The protein contains two conserved regions highly similar to the CHORD domains of different RAR1 proteins identified (**Figure 1**). Search of the wheat genomic DNA sequence database at the International Wheat Genomic Sequence Consortium (IWGSC) for homologs of *TaRAR1*, found five wheat contigs each contains a homolog of *TaRar1*, contig64299699 (7,236 bp) and contig4730984 (4,311 bp) from 2AL, contig7991159 (6,558 bp) from 2BL, and contig9718756 (1,215 bp) and contig9716294 (1,350 bp) from 2DL. The contigs from 2A and 2D chromosomes contain only partial sequence of the homolog. The full-length sequence was obtained through the joint of the overlapped sequence from the two contigs. In total, three homeologs of *TaRar1* have been identified in the wheat genomes, located on the long arms of chromosomes 2A, 2B, and 2D, thereafter referred to as *TaRar1-2A. TaRar1-2B*, and *TaRar1-2D.* The copy number and locations of the *TaRar1* homologs were further confirmed by a Southern hybridization using Chinese Spring nulli-tetrasomic lines (**Supplementary Figure S1**). The three encoded proteins in Chinese Spring share about 95–99% similarity with each other (**Supplementary Figure S2**). The sequence of Suwon11 *TaRar1* is the most similar to the nucleotides sequence of the Chinese Spring *TaRar1-2B*.

**Table 1:** Sequences of the primers used in this study.

Primer name	Primer sequences (5′–3′)
TaRar1-F	ATGTCGGCGGAGACGGAGAC
TaRar1-R	TCATACGGCATCAGCATTGTGC
TaEF-F	TGGTGTCATCAAGCCTGGTATGGT
TaEF-R	ACTCATGGTGCATCTCAACGGACT
Rar1-QRTF	AATAGGCTGCGACGCCATGT
Rar1-QRTR	GGTTTCTCAGTTGTATGCTTCCCT
Rar1-oligoF	CTGTTTCTCTTAACTCAAAGGCAACCCCACCAAAGTTAGCTCCAATCCAGTCTTCTAAGCAGGGTGTG GAAACCGAGGCCTGCTCCAGGTGCCGTCAGGG
Rar1-oligoR	GAGCATTTTTCGACAGGTTCCGTATTTGTACCATTTACAGCAACTGGTTTTTGTGCCTTCGGCTGTGAT CCATGGTCGGAGCAAAAGAAACCCTGACGGC
TaPR1_qRT-PCR_S	GAGAATGCAGACGCCCAAGC
TaPR1_qRT-PCR_AS	CTGGAGCTTGCAGTCGTTGATC
TaPR2_qRT-PCR_S	AGGATGTTGCTTCCATGTTTGCCG
TaPR2_qRT-PCR_AS	AAGTAGATGCGCATGCCGTTGATG
TaPR5_qRT-PCR_S	CAAGCAGTGGTATCAACGCAGAG
TaPR5_qRT-PCR_AS	GTGAAGCCACAGTTGTTCTTGATGTT
TaCAT_qRT-PCR_S	TGCCTGTGTTTTTTATCCGAGA
TaCAT_qRT-PCR_AS	CTGCTGATTAAGGTGTAGGTGTTGA
TaEF1-F	TGACCAGATCAACGAGCC
TaEF1-R	CTCCAGGAGAGACTCATG
PsEF-F	TTCGCCGTCCGTGATATGAGACAA
PsEF-R	ATGCGTATCATGGTGGTGGAGTGA


Alignments of the TaRAR1 with available RAR1 sequences of barley HvRAR1 (AF192261_1), rice OsRAR1 (XP_015623436.1), *Arabidopsis* AtRAR1 (BAB11239.1), and all the wheat alleles revealed highly conserved amino acid sequences at the two functional CHORD domains (**Figure 1**), implying RAR1 proteins from different plant species might have a similar function.

### *TaRar1* Transcriptional and SA Level Response to *Pst*

**FIGURE 2:**
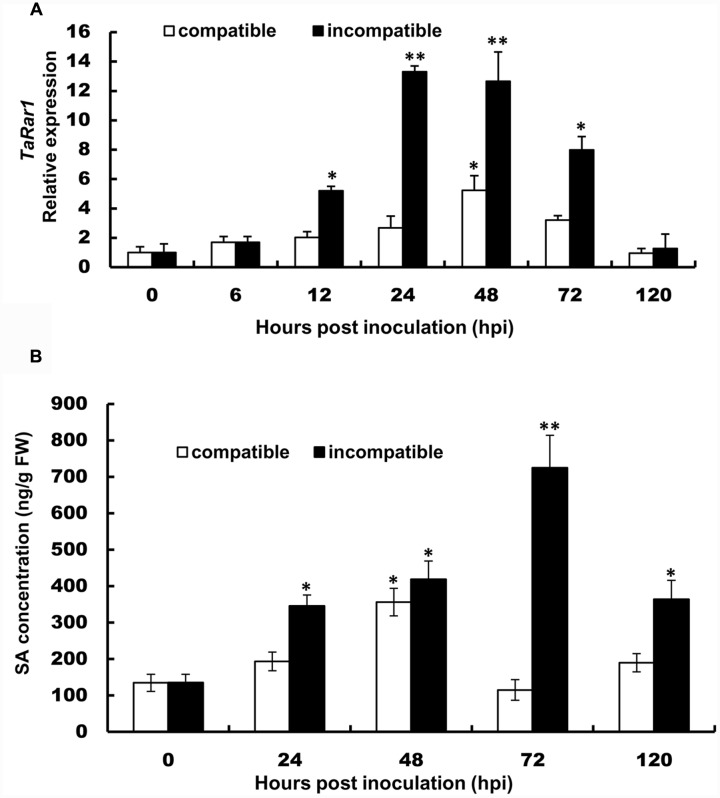
**Transcript levels of *TaRar1*** **(A)** and salicylic acid (SA) concentration **(B)** in Suwon11 leaves after inoculation with CYR23 (incompatible interaction) and CYR31 (compatible interaction). Leaf tissues were sampled at 0, 6, 12, 24, 48, 72, and 120 hours post inoculation (hpi). Three independent biological replications were performed. Relative gene expression was calculated by the comparative ΔΔCt method and was relative to the mock at each corresponding time point using gene-specific oligonucleotide primers (**Table 1**). Transcript abundance was normalized to the reference gene *TaEF-1a* (wheat elongation factor) (GenBank accession Q03033). The mean expression values were calculated from three replications. Error bars represent standard deviation. (^∗^) and (^∗∗^) indicate a significant difference between a particular hpi and 0 hpi with a *p*-value < 0.05 and 0.01, respectively. Differences were assessed using Student’s *t*-tests.

To study the expression profiles of the three *TaRar1* homeologs during wheat–*Pst* interactions, we challenged Suwon11 with two *Pst* pathotypes, CYR23 and CYR31. Suwon11 is resistant to CYR23, forming an incompatible interaction with the pathogen; and susceptible to CYR31, forming a compatible interaction with the pathogen. The transcript abundances of the three homeologs can’t be assayed separately due to the high similarity at the RNA level. Therefore, the expression of all three alleles were measured together (Referred to as a group as *TaRar1*) using conserved primers via quantitative real-time PCR (qRT-PCR). Expression was measured in samples generated from leaf tissues collected at seven time points post inoculation (**Figure 2A**). In both interactions, the total of three *TaRar1* homeologs expression was up-regulated starting from 6 h post inoculation (hpi), reached the highest level between 24 and 48 hpi and returned to the basal level (level of 0 hpi) at 120 hpi. The striking differences in the *TaRar1* expression between the two interactions were the timing and gratitude; the highest *TaRar1* level in the incompatible interaction was 24 h earlier and 13-fold stronger than that in the compatible interaction (**Figure 2A**). These results suggested that *TaRar1* might play a role in defense response against *Pst*.

The SA level was then measured in the two interactions at five time points (**Figure 2B**). SA level was significantly increased in both interactions during the course of pathogenesis. In the incompatible interaction, the earliest high SA level was detected at 24 hpi, peaked at 72 hpi and decreased at 120 hpi. The SA level was also increased significantly in the compatible interaction at 48 hpi, but was 12 h delayed than that in the incompatible interaction, similar to that observation in the *TaRar1* expressions. In addition, the high SA level in the compatible interaction only lasted for a short time, by 72 hpi, the SA level has declined to the basal level at 0 hpi (**Figure 2B**).

### *TaRar1* Transcript Level in Different Organs and Developmental Stages

**FIGURE 3:**
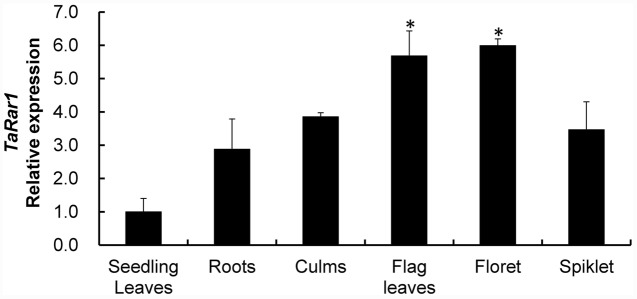
**Relative transcript levels of *TaRar1* in different wheat tissues.** Samples were taken from seedling leaves, roots, culms, flag leaves, floret, and spikelet. Three independent biological replications were performed. Relative gene expression was calculated by the comparative ΔΔCt method. Transcript abundances were normalized to the reference gene *TaEF-1a* and relative to that in seedling leaves (normalized as 1). Error bars represent standard deviation among three biological replicates. (^∗^) indicate a significant difference between a particular tissue and seedling leaves with a *p*-value < 0.05. Differences were assessed using Student’s *t*-tests.

*TaRar1* expression was also analyzed in six different tissues (roots, culms, seedling leaves, flag leaves, floret, and spikelet) collected from plants grown under normal condition. The gene expression pattern appeared to be tissue-specific. If normalizing the transcript abundance in the seedling leaves as a control, roots, culms and spikelet had about 2.8-fold, 3.8-fold, and 3.5-fold higher expression than the control (**Figure 3**), respectively. Floret and flag leaves had about sixfold higher than the control (**Figure 3**), suggesting higher *TaRar1* expression at the adult plant stage.

### *TaRar1* Expression in Response to Abiotic Stresses and Hormones

**FIGURE 4:**
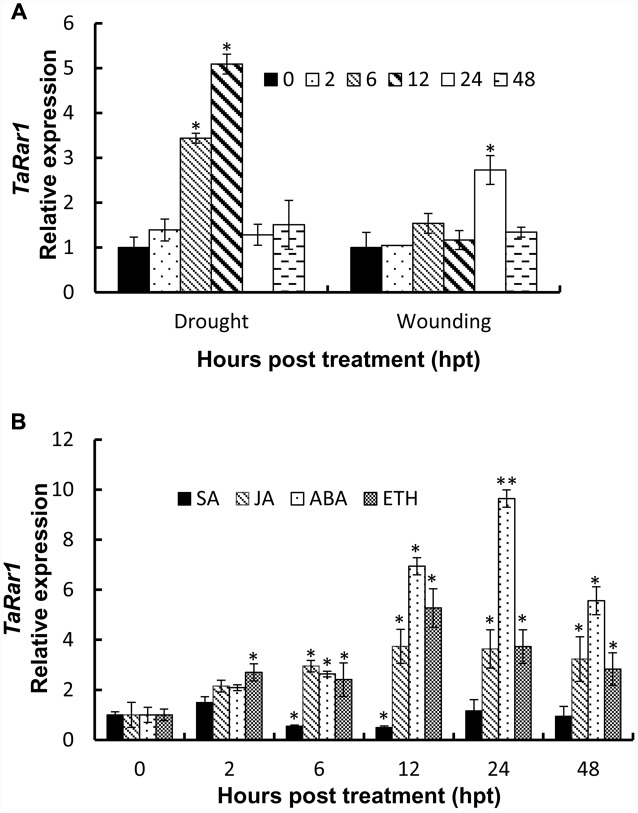
**Expression profiles of *TaRar1* in response to abiotic stresses** **(A)** and exogenous hormones **(B)**. Leaf tissues were sampled at 0, 2, 6, 12, 24, and 48 hours post treatment (hpt) both in wound and drought stresses **(A)**. Transcript abundances were normalized to the wheat elongation factor *TaEF-1a* gene and relative to the level at 0 hpi. Three independent biological replications were performed. Error bars represent standard deviation among three biological replicates. The asterisks (^∗^) and (^∗∗^) indicate a significant differences between that time point and 0 hpt with a *p*-value < 0.05 and 0.01, respectively. Differences were assessed using Student’s *t*-tests. ABA, abscisic acid; SA, salicylic acid; ET, ethylene; MeJA, methyl jasmonate.

To study *TaRar1* in response to abiotic stress, we treated plants with PEG6000 to induce drought stress and poked leaves by a sterilized scissors to cause wounding. *TaRar1* transcription level was measured at five time points as shown in **Figure 4**. Real-time PCR revealed a rapidly induction of *TaRar1* under drought stress as early as 6 h post PEG6000 treatment (hpt), and the highest fivefold increase at 12 hpt (**Figure 4A**), then restored to the 0-hpt control level at 24 hpt. Similarly, *TaRar1* expression was also increased after wounding, but the induction was relatively slower compared to the PEG treatment, a significant threefold increase was detected at 24 hpt, and then backed to the control level by 48 hpt (**Figure 4A**).

To understand the *TaRar1* regulation by plant hormones, we treated leaves with four different plant hormones exogenously; including SA, JA, abscisic acid (ABA), and ethanol (ET) at the two leaves stage. Leaf tissues were collected at six different time points (**Figure 4B**). The 0 hpt level was normalized as 1 for each treatment. As shown in **Figure 4B**, when treated with SA, *TaRar1* was either unchanged or significantly reduced during 6–12 hpt. In contrast, *TaRar1* was significantly up-regulated after treating with JA, ABA, or ET. The highest 10-fold of that of the 0-hpt level *TaRar1* expression was detected at 24 h post-ABA treatment. The *TaRar1* induction by JA or ET treatment was not as high as that detected in the ABA treatment, but all was significantly higher than the control level at 0 hpt.

### Down-Regulating *TaRar1* Compromised Wheat Resistance to an Avirulent Strain of Stripe Rust

Because the higher *TaRar1* expression was associated with the incompatible interaction, we investigated if down-regulating *TaRar1* expression would compromise Suwon11 resistance to the avirulent *Pst* pathotype. We knocked down the endogenous *TaRar1* transcripts in Suwon11 using a barley stripe mosaic virus induced gene silencing (BSMV-VIGS) assay targeting all three *TaRar1* homeologs using a 190-bp highly conserved region (**Supplementary Figure S3**), the vector is designed as BSMV:Rar1. Two constructs carrying only the BSMV genomes and a 120-bp wheat *phytoene desaturase* (*PDS*) gene were included as controls in the study, named as BSMV:00 and BSMV:PDS. In addition, a mock control only inoculated with the FES buffer was also included.

**FIGURE 5:**
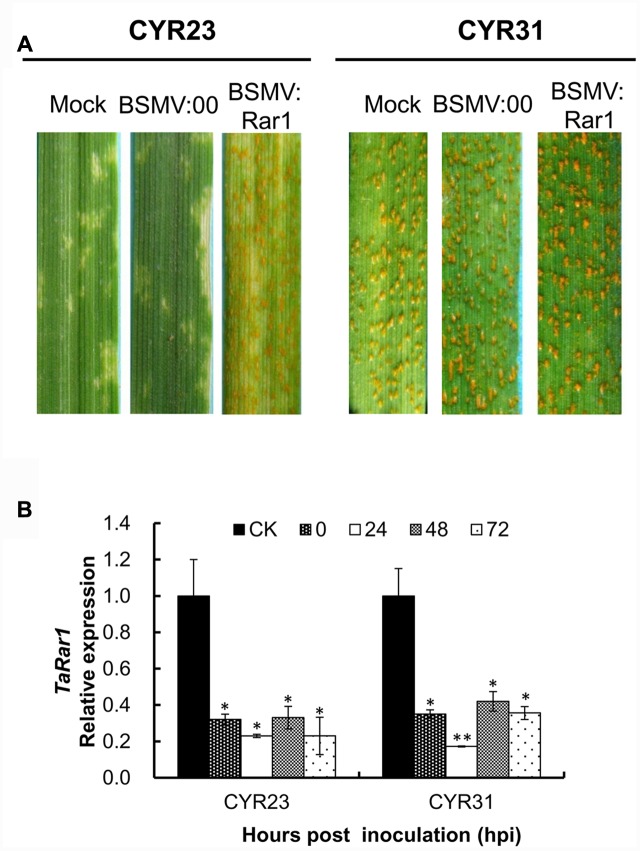
**Functional analysis of the *TaRar1* gene by the BSMV-induced gene silencing assay.** **(A)** Infection type of Suwon11 to CYR23 (avirulent pathotype) and CY31 (virulent pathotype) after inoculated with BSMV:00 and BSMV:Rar1. Stripe rust was inoculated nine days post BSMV inoculation. Pictures were taken 15 days post rust inoculation. Mock: wheat leaves inoculated with FES buffer. BSMV:00 means only the BSMV genome; BSMV:Rar1 means a 190-bp fragment of TaRar1 was inserted into the BSMV gamma genome. **(B)** Transcript abundances of *TaRar1* in Suwon11 during the course of rust infection. *X*-axis indicates the time post inoculations (hpi) by two *Pst* pathotypes at 0, 24, 48, and 72 h. CK: Suwon11 treated with BSMV:00. Different patterns of bars indicate different time points post rust inoculations. Relative expressions of *TaRar1* were normalized to the CK at 0 hpi (as 1) in each of the rust inoculations. Error bars represent the variations among three independent replicates. (^∗^) and (^∗∗^) indicate a significant differences between that time point and CK with a *p*-value < 0.05 and 0.01, respectively.

Mild chlorotic mosaic symptoms were appeared after BSMV-inoculated plants at 5–8 dpi, and no distinct defects on newly emerged leaves were observed. At 9 dpi, photobleaching was displayed on the plants inoculated with BSMV:PDS (data not shown), indicating BSMV induced gene silencing was started. Two *Pst* pathotypes CYR23 and CYR31 were inoculated the *TaRar1* silenced plants, respectively. Suwon11’s resistance level described as infection types (ITs) was scored at 15 dpi. As shown in **Figure 5A**, Suwon11 showed a resistant response after inoculated with CYR23 on the mock and BSMV:00 controls, characterized with a high necrosis areas at the infection sites. *TaRar1* silenced leaves had more fungal sporulation than the mock and the BSMV:00 controls (**Figure 5A**, left), suggesting down-regulation of *TaRar1* compromised the Suwon11 resistance to the avirulent pathotype CYR23. When inoculated with the virulent CYR31 pathotype, *TaRar1* silenced leaves had the same IT as the controls (**Figure 5A**, right).

Leaf tissues were collected to ensure the effective silencing assays from both BSMV:Rar1 and BSMV:00 plants right before *Pst* inoculations labeled as 0 hpi, and then 24 hpi, 48 hpi, and 72 hpi. The transcription level of *TaRar1*was knocked down by approximately about 70–80% in the BSMV:Rar1 silenced leaves compared to the leaves of the same growth stage from BSMV:00-infected plants, labeled as CK in **Figure 5B**. The results confirmed the silencing of *TaRar1* before and during *Pst* infection.

### Endogenous SA Level Decreased in *TaRar1* Silenced Leaves

**FIGURE 6:**
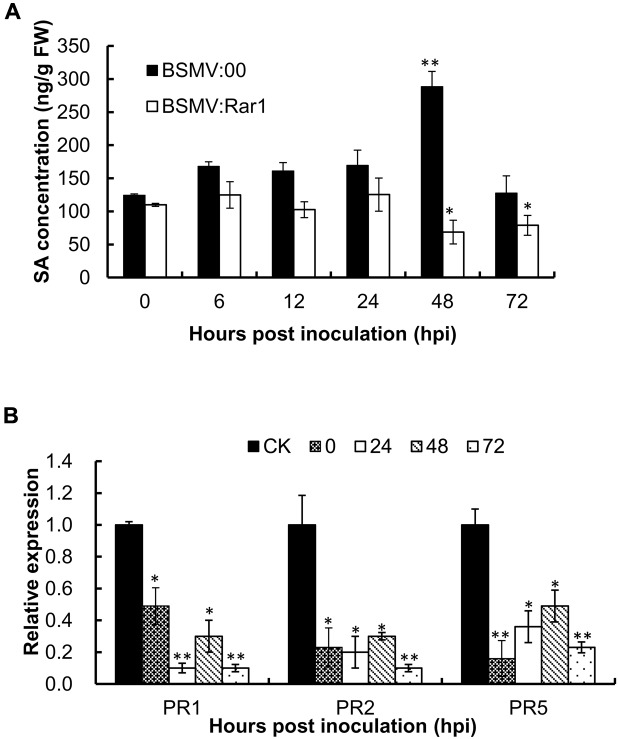
**Salicylic acid concentration** **(A)** and *PR* gene expression **(B)** in *TaRar1* silenced leaves during the time course of stripe rust infection. Sample tissues were collected at 0, 6, 12, 24, 48, and 72 hhpi challenged with CYR23 *Pst* pathotype. Three independent biological replications were performed. Transcript abundance was normalized to the reference gene *TaEF-1a* (wheat elongation factor) (GenBank accession Q03033). The mean expression values were calculated from three replications. Error bars represent standard deviation among three biological replicates. (^∗^) and (^∗∗^) indicate a significant difference between a particular hpi and CK with a *p*-value < 0.05 and 0.01, respectively. Differences were assessed using Student’s *t*-tests.

To analyze whether the endogenous concentration of SA was affected by the *TaRar1* expression, we measured the SA level in BSMV:Rar1 silenced leaves and controls after the avirulent *Pst* inoculation at six time points (**Figure 6A**). In the BSMV:00 control plants, the SA levels were up-regulated and peaked at 48 hpi. However, the concentrations of SA were almost unchanged or reduced over the time course and had significantly lower expression than the control (BSMV:00) from 12 to 72 hpi (**Figure 6A**). In addition, three *PR* genes *PR1. PR2*, and *PR5* were monitored during the same time course study. qRT-PCR revealed that all three *PR* genes had significantly low expressions when *TaRar1* was silenced compared to the non-*TaRar1* silenced control CK in **Figure 6B**.

### Exogenous SA, JA, or ABA Treatment Enhanced Wheat Resistance to a Virulent Strain of Stripe Rust

Our studies have revealed that high *TaRar1* expression after exogenous treatment of JA or ABA, and higher endogenous SA was associated with higher *TaRar1* expression in the incompatible interaction. Therefore, we tested the level of endogenous SA and the wheat defense response after treatment with JA or ABA. As shown in **Supplementary Figure S4A**, endogenous SA concentration was increased as early as 12 h post ABA treatment (hpt) and with as much as eightfold increase at 24 hpt. A transient high SA level was detected post JA treatment at 24 hpt. In addition, Suwon11 showed less sporulation by the virulent CYR31 strain after exogenous SA, JA, and ABA treatments (**Supplementary Figure S4B**). The findings were confirmed by the less pustule counts per area (**Supplementary Figure S4C**) and relative lower fungal DNA concentration in the leaves pre-treated with a hormone compared to the control (**Supplementary Figure S4D**).

### Silencing of *TaRar1* Increased Fungal Growth and Reduced Cell Death

**FIGURE 7:**
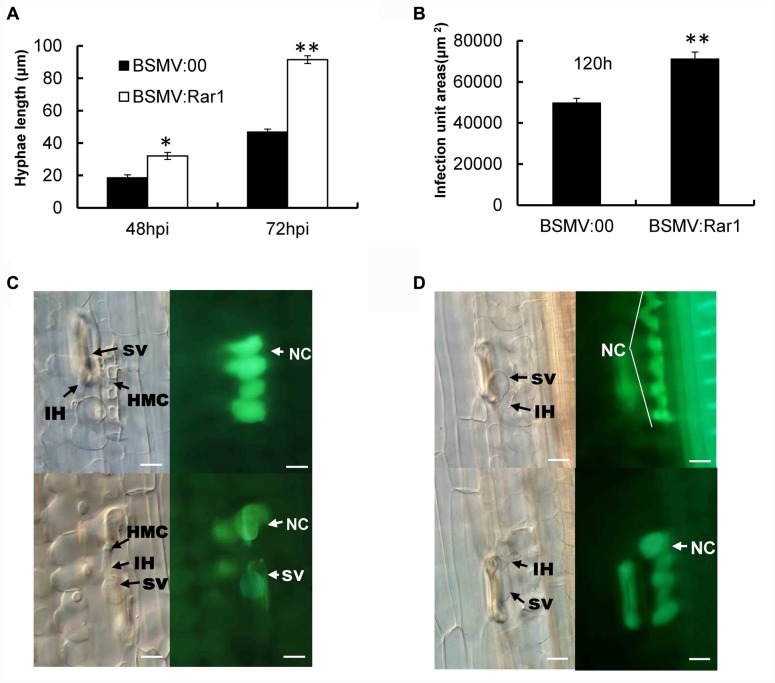
**Pathotype CYR23 growth on Suwon11 measure by hyphae length** **(A)**, infection areas at 120 hpi **(B)** and cell death in Suwon11 leaves at 48 hpi **(C)** and 72 hpi **(D)**. Suwon11 was inoculated with BSMV:00 or BSMV:Rar1 and then was inoculated with CYR23 nine days post inoculation. **(A)** Hyphal length was measured under a light microscope at 48 and 72 h post CYR23 inoculation in both *TaRar1*-silenced and non-silenced plants. The growth of hyphae was significantly increased in *TaRar1*-silenced Suwon11. **(B)** Infection areas were measured microscopically at 120 hpi in both TaRar1-silenced and non-silenced Suwon11. Infection area was significantly enlarged in *TaRar1* silenced leaves. **(C,D)** Histological observations of cell death. Pictures were taken under an epifluorescence or light microscopy at 48 hpi **(C)** and 72 hpi **(D)**. Significant reduced green florescence in the necrotic area per infection size in *TaRar1*-silenced plants. NC, necrotic cell; SV, substomatal vesicle; IH, initial hyphae; HMC, haustorial mother cell; Bars = 50 μm. Values represent mean ± standard errors of three independent samples. Differences were assessed using Student’s *t*-tests. (^∗^) and (^∗∗^) indicate a significant difference between a particular hpi and CK with a *p*-value < 0.05 and 0.01, respectively.

*TaRar1* silenced Suwon11 leaves were examined microscopically after inoculation with the avirulent pathotype CYR23 to determine any histological changes associated with the enhanced susceptibility. Fungal development and host responses to CYR23 were similar to what have been described previously (Wang et al., 2008, 2013). At 48 hpi and 72 hpi after inoculated with CYR23, the fungal hyphae were significantly (^∗^*P* < 0.05, ^∗∗^*P* < 0.01) longer in the *TaRar1* silenced leaves compared to that in the leaves of BSMV:00 inoculated control (**Figure 7A**). Meanwhile, the total infection area was significantly larger (^∗∗^*P* < 0.01) in the *TaRar1*-knockdown plants at 120 hpi relative to the control (**Figure 7B**).

Hypersensitive cell death evidenced by the yellow auto-florescence under a florescence microscope was documented at 48 and 72 hpi and compared between *TaRar1* silenced leaves and the control. The auto-fluorescence of attacked mesophyll cells were observed under an epifluorescence microscopy with excitation filter, 485 nm; dichromic mirror, 510 nm; and barrier filter, 520 nm. The yellow color is changed to green because of the settings of saturability and the contrast of light. The control leaves at the infection sites showed stronger auto-florescence compared to the *TaRar1* silenced leaves at 48 hpi (**Figure 7C**) and 72 hpi (**Figure 7D**), suggesting that silencing of *TaRar1* reduced the degree of cell death and resulted in more fungal growth.

### *TaRar1* Was Involved in Reactive Oxygen Accumulation Process

**FIGURE 8:**
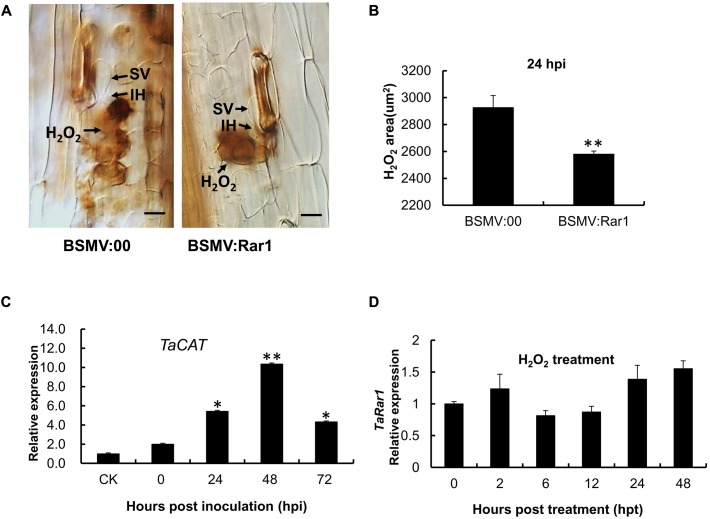
**Reactive oxygen accumulation and related gene expression in the *TaRar1* knockdown plants.** **(A)** Histological observation of H_2_O_2_ accumulation (Brown color) by DAB stain in Suwon11 leaves 24 h post CYR23 inoculation in BSMV:00 and BSMV:Rar1 inoculations leaves. H_2_O_2_ accumulation was significantly reduced in *TaRar1* silenced leaves compared to the control. SV, substomatal vesicle; IH, initial hyphae; Bars = 50 μm. **(B)** DAB stained areas measured microscopically at 24 hpi. **(C)** The expression level of *TaCAT* catalase (X94352) after BSMV:Rar1 inoculation at four time points relative to the expression in BSMV:00 (CK). **(D)** *TaRar1* expression profile after exogenous H_2_O_2_ treatment. Transcript abundance of *TaRar1* had no obvious change at different time points post treatment. (^∗^) and (^∗∗^) indicate a significant difference between a particular hpi and CK with a *p*-value < 0.05 and 0.01, respectively.

Because hydrogen peroxide (H_2_O_2_) has been associated with HR, we were interested to find out any changes of H_2_O_2_ accumulation in *TaRar1* silenced leaves upon pathogen challenge. We used diaminobenzidine (DAB) polymerization to show H_2_O_2_ accumulation in infected leaf tissues *in situ*. Interestingly, similar to the control, BSMV: Rar1 inoculated plants had H_2_O_2_ accumulation in mesophyll cells at 24 hpi with the avirulent CYR23 pathotype (**Figure 8A**). However, the striking difference was that H_2_O_2_ accumulation was significantly (^∗∗^*P* < 0.01) reduced in the *TaRar1* silenced leaves compared to the control (**Figure 8B**). In contrast, the expression of the wheat catalase gene (*TaCAT*), which is involved in ROS removal, was significantly increased (^∗^*P* < 0.05, ^∗∗^*P* < 0.01) in the *TaRar1*-knockdown leaves after infection with CYR23 (**Figure 8C**), implying that the reduction of H_2_O_2_ accumulation was the result of higher expression of *TaCAT*. Real-time PCR revealed little change in *TaRar1* transcript level when treated wheat plants with exogenous H_2_O_2_ (100 mM) (**Figure 8D**). These results suggested that *TaRar1* functions upstream of H_2_O_2_ accumulation in responses to *Pst* infection.

## Materials and Methods

### Plant Materials

Wheat (*T. aestivum* L.) cultivars Suwon11 (spring wheat) and two stripe rust *Pst* pathotypes CYR23 and CYR31 were the biological materials used in this study. Seedlings of Suwon11 was grown and maintained followed the procedure described by Kang and Li (1984). Suwon11 has been reported containing the stripe rust-resistance gene *YrSu* and highly resistant to CYR23 but highly susceptible to CYR31 (Stakman et al., 1962). Chinese Spring nulli-tetrasomic lines were kindly provided by the Wheat Genetics and Genomics Center at Kansas State University, Manhattan, KS, USA.

### Cloning of *TaRar1* and Sequence Analysis

To clone the *TaRar1 g*ene, a pair of primers (forward and reverse) was designed using Primer 5.0 software. The primers were designed based on the cDNA sequence from our laboratory database, which was obtained from the interaction between the Suwon11 cultivar and CYR23 (avirulent) or CYR31 (virulent) (**Table 1**). The primers were used to amplify the open reading frame (ORF) of *TaRar1*. The template was a mixture of the first strand cDNA samples extracted from leaves of Suwon11 at 12, 24, 48, and 72 h post inoculated with CYR23 (an incompatible combination). The PCR products were cloned into the pGEM-T Easy Vector System (Promega, Madison, WI, USA) or the pMD18-T Simple Vector (TaKaRa Biotechnology)^1^ and sequenced using an ABI PRISM 3130XL Genetic Analyzer (Applied BioSystems). The amino acid sequence of TaRar1 was analyzed to determine the alignment of the deduced protein sequences using the DNAMAN (version 6) program (Lynnon Biosoft, Quebec, Canada).

### Nulli-tetrasomic Analysis via Restriction Fragment Length Polymorphism (RFLP)

For RFLP, enzyme digestion, gel-electrophoresis, Southern blotting, probe, labeling, and hybridization were performed following the protocols described by Huang et al. (2003). A 200-bp DNA fragment from the CHORD-I region was used as a probe. Genomic DNA of CS nulli-tetrasomic lines were digested with *Eco*RV.

### RNA Isolation and Real-Time PCR

Leaf tissues for time course study were collected at the corresponding time points from different plants. For tissue-specific expression analyses of *TaRar1*, intact seedling leaves were sampled at the two-leaf stage. The same plants then were used for root, stem, flag leaf, floret, and spikelet tissues at the adult stage at the same time. Sampled tissues were flash-frozen in liquid nitrogen and stored at -80°C before the isolation of total RNA. Each experiment has three independent biological replications included.

The RNA isolation was conducted with Biozol^TM^ Reagent (BioFlux, Tokyo, Japan). The integrity and quality of the total RNA was characterized in a 1% agarose gel. Additionally, A NanoDrop^TM^ 2000 spectrophotometer (Thermo Fisher Scientific, USA) was used to estimate the RNA quantity. A Revert Aid First-strand cDNA synthesis kit from Fermentas^2^ was used to synthesize cDNA from RNA.

Real-time PCR reactions and primer design were performed as described by Wang et al. (2013). A CFX connect^TM^ Real-Time PCR System was used to perform the quantitative real-time PCR (Applied Biosystems, Foster City, CA, USA). *TaEF-1a* gene (GenBank accession number Q03033), a wheat elongation factor was used as a housekeeping reference for real-time PCR analysis. An 85-bp *TaEF-1a* fragment and a 190-bp TaRar1 fragment as gene specific primers are synthesized to determine the gene expression, listed in **Table 1** as TaEF-F/R and TaRar1-QRTF/QRTR. Specific amplification was contacted to ensure for each reaction according to a single peak dissociation curve. The comparative 2^-ΔΔCT^ method was used to quantify relative gene expression according to the Threshold values (*C*t) generated from Biosystems applied (Livak and Schmittgen, 2001).

### Rust Inoculations and Chemical Treatments

Freshly stripe rust urediniospores were brushed to the surface of primary wheat leaves at 7-day-old seedlings. The control treatment was inoculated with sterile distilled water. Plants were incubated for 24 h in dark in a dew chamber with a temperature of 15°C and 100% humidity. Then the plants were subsequently transferred to a growth chamber with a temperature of 16°C and a 16 h photoperiod. Leaves were collected at 0, 6, 12, 24, 48, 72, and 120 hpi. These time points were related with a series of biological events in the interactions between Suwon11 and CYR23 or CYR31 (Wang et al., 2013).

Different chemical treatments were sprayed to the leaf surface of wheat seedlings with 20 mM SA, 2 mM methyl jasmonate (MeJA), 2 mM ABA, 2 mM ethylene (ET) and 100 mM H_2_O_2_, respectively (Wang et al., 2013). All chemicals were dissolved in 0.1 % (v/v) ET. The mock plants were sprayed with 0.1% (v/v) ET.

### Analyses of the Expression of *TaRar1* in Response to Different Abiotic Stress Treatments

To analyze the expression of *TaRar1* under drought and wounded conditions, 30 wheat seedlings were prepared for each treatment. For the drought-stress treatment, roots of wheat seedlings were soaked in 200 mM NaCl or 20% PEG6000. The mock-treated seedlings were maintained in a growth chamber at a normal temperature with a 12 h photoperiod. The wound treatment was applied by cutting the wheat leaves using sterilized scissors. Leaf tissues were sampled at 0, 2, 6, 12, 24, and 48 hpt and rapidly frozen in liquid nitrogen and stored at -80°C. Three independent biological replicates were used for each time point and control.

### BSMV-Mediated Gene Silencing

Method for constructing the silencing plasmids was conducted according by Holzberg et al. (2002). A 120-bp cDNA fragment was amplified from the wheat *PDS* gene *TaPDS* by reverse-transcriptase polymerase chain reaction (RT-PCR). BSMV:GFP (green fluorescent protein) cDNA as the starting material was used to create BSMV:PDS. A PDS fragment replaced the GFP coding sequence in BSMV:GFP resulting in BSMV:PDS. The same approach was constructed to get BSMV:Rar1, a 190-bp *TaRar1* cDNA fragment amplified by the primers TaRar1-oligo-F/R(**Table 1**).

Tripartite BSMV genome was linearized and transcript to RNA *in vitro* (Holzberg et al., 2002) using the mMessage mMachine T7 *in vitro* transcription kit (Ambion, Austin, TX, USA). The BSMV inoculum was made by combining an equimolar ratio of α, β, and γ transcripts at a 1:1:1 ratio mixed with inoculation buffer (named as FES) containing a wounding agent. The inoculation was done on the second leaf of three-leaf stage seedling with BSMV RNA by gently rubbing the leaf surface with a gloved finger. Three independent sets of inoculations were performed, with a total of 72 seedlings inoculated for each of the three BSMV viruses (BSMV:00, BSMV:PDS, and BSMV:Rar1). Twenty-four seedlings inoculated with 1xFES buffer were included as a negative control. Post viral inoculation, wheat plants were maintained in a growth chamber at 25 ± 2°C, and examined for symptoms at regular intervals. Once photobleaching was observed, three independent sets of inoculations including CYR23, CYR31 and sterile water (as a mock), were performed. ITs of stripe rust were examined at 15 days post rust inoculation. The third leaves corresponding to the photobleached areas of BSMV: PDS infected plants were divided at 0, 24, 48, and 120 hpi for histological observation and real-time PCR assay. The primers used to assay the transcript abundances of the *TaPR1. TaPR2. TaPR5*, and *TaCAT* genes in the *TaRar1*-knockdown wheat seedlings are listed in **Table 1**.

### SA Level Analysis with the HPLC-MS

Leaves were collected and immediately frozen in liquid nitrogen. The extraction of SA was according to Segarra et al.’s (2006) method and modified as followed. Frozen samples were then ground under liquid N_2_ with mortar and pestle. An amount of 200 mg of the resulting powder was extracted with 750 μl MeOH–H_2_O–HOAc (90:9:1, v/v/v) and centrifuged for 1 min at 10,000 rpm. The supernatant was collected and the extraction was repeated twice. Pooled supernatants were dried under N2, resuspended in 1000 μl of pure chromatographic grade MeOH, and finally filtered with a Millex-HV 0.22 μm filter from Millipore (Bedford, USA). Quantitation was done by the standard addition method by spiking control plant samples with SA solutions (ranging from 50 to 1000 ng ml^-1^), and extracting as described above.

### Histological Observation of Fungal Growth and ROS Accumulation

Wheat leaves were sampled at 48 and 72 hpi with *Pst* after inoculated with BSMV and treated as Wang’s methods (Wang et al., 2013). Cleared leaf segments were observed using an Olympus BX-51 microscope (Olympus Corp., Tokyo) for infection areas and lengths of infection hyphae. Infection areas were the areas containing infection hypha at an infection unit. An infection unit is a site when intercellular fungal mycelium in the leaf mesophyll cell layer is formed by a germling that penetrated through a stoma. Auto-fluorescence was observed as a necrotic death area in infected mesophyll cells by epifluorescence microscopy. More than 30 infection sites were chosen to examine the auto-fluorescence on each of five randomly selected leaf segments per treatment. The infection sites were considered as successfully penetrated with fungal appressoria formation over stomata. The necrotic area was measured with a calibrated eyepiece micrometer and corresponding necrotic areas (square micrometers) calculated as Wang’s method (Wang et al., 2013). SPSS software was used to statistical analysis the standard deviations and Tukey’s test (SPSS, Inc. Chicago, IL, USA).

In order to study the host response, H_2_O_2_ accumulation was detected as described by Thordal-Christensen et al. (1997) in plant mesophyll cell. Cutting the inoculated wheat leaves and immersing the ends in a stained buffer containing 1 mg/ml 3,3′-DAB dissolved in HCl-acidified (pH 3.8) distilled water. Leaves were incubated for 8 h in the DAB buffer and transferred to the fixed buffer to terminate the reaction.

### Relative Quantification of *Pst* in Inoculated Leaves

The single-copy target genes *PsEF* and *TaEF1* were used to measure the relative quantification of *Pst* as carried out (Panwar et al., 2013). Standard curves were prepared with the genomic DNA of the wheat cultivar Suwon11 and the *Pst* pathotype CYR31 at seven serial dilutions, respectively. The correlation coefficients for the standard curves were above 0.99. The specific primers PsEF-F/R and TaEF1-F/R were used to do the quantification PCR listed in **Table 1**. The biology biomass of the real-time PCR products of *PsEF* and *TaEF1* in infected sample leaves were calculated according to the gene-specific standard curves to analysis the quantification of *Pst* and wheat genome DNA.

## Discussion

In this study, we cloned and characterized a *TaRar1* gene from cultivar Suwon11. The gene has 14 polymorphisms at the nucleotide level but only three amino acid differences at the protein level (**Figure 1** **Supplementary Figure S5**) compared to the *TaRAR1-1* amplified from another wheat cultivar WGRC7 (Tai, 2008). Three *TaRar1* homologs obtained from the wheat cultivar Chinese Spring genomic DNA sequence at the IWGSC share 94–99% similarity with each other (**Supplementary Figure S3**), and locate at wheat homeologous group 2 chromosomes 2A, 2B, and 2D (**Supplementary Figure S1**), therefore they were referred as homeologs. It is highly possible that three *TaRar1* homeologs exist in Suwon11 as well. Since the three homeologs were knocked down simultaneously and measured together in this study, we drew the conclusion using *TaRar1* to represent the function of the three homeologs.

RAR1 protein contains two CHORD domains rich in cysteine and histidine. The domains are involved in zinc-dependent protein–protein interactions. Comparison among RAR1 from different species or different cultivars of the same species revealed that the two functional domains of CHORD-I and CHORD-II are highly conserved (**Figure 1**; **Supplementary Figure S2**), suggesting an important and also similar function associated with the CHORD domains of the RAR1 protein from different species. It has been shown that the RAR1 CHORD-I interacts with the CS (CHORD-containing protein and SGT1) domain of SGT1 and CHORD-II interacts with M domain of HSP90 to form a complex. The protein complex functions as a chaperone complex for NLR immune sensors (Tornero et al., 2002; Bieri et al., 2004; Holt et al., 2005; Azevedo et al., 2006) to modulate downstream defense responses including HR and *PR* gene expressions. The function of RAR1 is conserved in different plant species, containing *Arabidopsis*, tobacco, and barley, and it is required by the subsets of both CC-NB-LRR and TIR-NB-LRR-type R proteins (Shirasu and Schulze-Lefert, 2003). In tobacco, silencing *Rar1* by virus-induced gene silencing strongly reduced the resistance to tobacco mosaic virus mediated by the *N* gene (Liu et al., 2002). Similarly, silencing three *TaRar1-1* homeologs in WGRC7 compromised the leaf resistance mediated by *Lr21* (Scofield et al., 2005). HvRar1 was first identified as a requirement for *Mla12* mediated resistance to powdery mildew. However, in the same species, mediating resistance to the same powdery mildew pathogen, but not all *Mla* genes need *HvRar1* in the pathway (Azevedo et al., 2002). Similarly, leaf rust resistance gene *Lr21* and stem rust resistance gene *Sr33* are both CC-NBS-LRR type of *R* gene in wheat (Huang et al., 2003; Periyannan et al., 2013), *TaRar1* is involved in the *Lr21* (Scofield et al., 2005) but not in the *Sr33*-mediated resistance (Periyannan et al., 2013). Generalizing from the studies on *Rar1* gene, it is clear that not all *R* genes with similar structures require *Rar1* during defense response. This suggests a fine-tuning of defense response signaling mediated by each *R* gene. This also implies a different satiability of each R protein since a known function of RAR1-SGT1-HSP90 complex is protein chaperon. Comprehensive understanding of each *R* gene-mediated signaling pathway will provide a key to this puzzle. In our study, down-regulating three homeologs of *TaRar1* in Suwon11 reduced the host resistance level to the avirulent *Pst* strain CYR23, suggesting this gene is required in the resistance to *Pst* infection mediated by *YrSu* in Suwon11.

In many cases, race-specific resistance is characterized by a rapid development of HR at the infection sites. Compared to the compatible interactions, we found the transcript level of *TaRar1* was strikingly up-regulated at the early stages of the pathogen infection (**Figure 2A**) in incompatible interaction. When *TaRar1* was silenced, the HR areas (**Figures 7C,D**) and production of H_2_O_2_ (**Figures 8A,B**) were significantly reduced, suggesting the *TaRar1* gene was required for inducing HR and ROS. SA has been shown to be a signaling molecule involved in both local HR and production of ROS at the infection sites and SAR to further infection by broad range pathogens (Durner and Klessig, 1995). In the incompatible interaction, SA concentration in Suwon11 was significantly increased as early as 24 hpi (**Figure 2B**). Down-regulating *TaRar1* reduced SA accumulation and HR compared to the control (**Figure 6A**), suggesting *TaRar1* might modulate defense response through signaling molecule SA. This hypothesis was supported by the observation that the disease severity by the virulent strain on Suwon11 was reduced after exogenous SA treatment (**Supplementary Figures S4B–D**). However, interestingly, the expression of *TaRar1* was reduced after exogenous SA treatment (**Figure 4B**). Notably, during the early infection course of pathogenesis, both *TaRar1* and SA were up-regulated (**Figures 2A,B**), but the timing of the induction seemed to suggest a higher *TaRar1* expression leaded to a higher SA accumulation. However, when SA level reached the peak at 72 hpi, *TaRar1* level has dropped compared to its highest level at the same time point (**Figures 2A,B**). These results suggested a negative feedback regulation of SA to *TaRar1* in this regulation, when SA accumulation exceeding a certain threshold level or exogenous high SA level was applied, *TaRar1* expression started to reduce (**Figure 4B**). Because SA level started to drop once *TaRar1* was reduced, suggesting *TaRar1* functions upstream in the SA biosynthesis pathway. These results suggested *TaRar1* involved in defense response against *Pst* through SA in the incompatible interaction (**Figures 2A,B**). Application of JA/ET and ABA also up-regulated *TaRar1* and enhanced Suwon11 resistance to the virulent strain (**Supplementary Figures S4A–D**), suggesting *TaRar1* involved in both race-specific defense and basal defense responses to *Pst*. These results are similar to those observed in the *AtRar1* mediated pathogen-associated molecular pattern-trigged immunity (PTI) in the absence of the cognate resistance gene in *Arabidopsis* (Shang et al., 2006), and *HvRar1*-mediated basal resistance to *Magnaporthe grisea* in barley (Jarosch et al., 2005). The induction of PTI in *Arabidopsis* mediated by AtRAR1 was through JA signaling and the interaction with SGT1 (Shang et al., 2006).

Reactive oxygen species are thought to play key roles in defense responses, in which the most important component of ROS is H_2_O_2_ (Keppler et al., 1989; Hemetsberger et al., 2012). We previously studied the generation and accumulation of ROS in the interactions of Suwon11 and two races of *Pst* (avirulent and virulent). In the incompatible interaction, H_2_O_2_ was detected at 12 hpi and percentage of infection sites showing H_2_O_2_ accumulation further increased until 24 hpi, which coincided with primary haustoria formation in mesophyll cells (Wang et al., 2008). At these different time points, the transcript levels of *TaRar1* were up-regulated compared with the control (**Figure 2A**). To analyze whether *TaRar1* affects ROS accumulation in wheat, we measured the production of H_2_O_2_ after knocking down *TaRar1* at 24 hpi. In control BSMV: 00 plants, abundant ROS accumulation were exhibited in a few mesophyll cells (**Figure 8A**, left). In contrast, mesophyll cells in contact with primary hyphae showed less ROS accumulation in BSMV: Rar1-silenced plants compared to the control (**Figure 8A**, right). The same event was observed in *Mla12*-triggered and *Rar1*-dependent oxidative burst, coinciding with fungal haustorium differentiation (Shirasu et al., 1999). SA radicals, which are generated from SA, binds and inhibits catalase which is function as a major H_2_O_2_-scavenging enzyme, thereby leading to an increase in the endogenous level of H_2_O_2_ (Durner and Klessig, 1995). We assayed the expression of catalase, which directly correlated with less SA accumulation, was upregulated after silencing *TaRar1* at 48 hpi (**Figure 8C**). The results were consistent with the reduced H_2_O_2_ accumulation after knocking down *TaRar1*, suggesting *TaRar1* activated the defense against *Pst* through modulating the H_2_O_2_ accumulation by SA signaling.

In addition to the involvement in defense against *Pst*, high level of *TaRar1* transcript abundance was also detected in wheat tissues at the adult stage (**Figure 3**), under drought or wounding stresses (**Figure 4A**), and after treatment with three out of four tested hormones (**Figure 4B**). The result implied a cross-talk through *TaRar1* among different signaling pathways modulating plant development and different stresses. The highest *TaRar1* level was seen in response to exogenous ABA treatment. The role of ABA in plants is complicated and our knowledge on ABA regulation is incomplete yet. However, studies on this plant hormone have demonstrated that ABA plays an ambivalent role in plant defense response depending on the timing of the infection and the interaction of the host and pathogen pair. ABA is a global switch in modulation signaling pathways overlapped among plant development, biotic and abiotic stresses (Asselbergh et al., 2008). In our studies, among the four hormones tested, *TaRar1* level was up-regulated after treatment with ABA, JA, or ET. An elevated SA level was seen after high level of *TaRar1* (**Figure 2A**). The positive effect of JA and ABA on SA level was confirmed by the high SA concentration detected after exogenous JA or ABA treatment (**Supplementary Figure S4A**). Similarly, a cooperative or synergistic interaction between SA and JA/ET was reported in several other studies (Xu et al., 1994; Mur et al., 2006) although antagonistic interaction between JA/ET and SA was also well documented (Audenaert et al., 2002; Ellis and Turner, 2002; Spoel et al., 2003; Xu et al., 2013). *Rar1* is a single copy gene in barley (Azevedo et al., 2002), *A. thaliana* (Muskett et al., 2002; Tornero et al., 2002), and potato (Pajerowska et al., 2005). However, in wheat, there are three highly conserved homeologs located on 2A, 2B, and 2D chromosomes, respectively (**Supplementary Figure S3**). With the VIGS strategy and real-time used in this study, we were unable to study the function and expression of each individual homeolog because of the highly similar DNA sequence among them. More investigation is needed to determine whether and how *TaRar1* connects different signaling pathways, is a homeolog-dependent or a dosage-dependent. Knockout mutation on each individual homeolog via EMS or fast-neutron would make the functional study of each homeolog possible to answer the above questions.

## Conclusion

We propose a working model, in which *TaRar1* was placed in the upstream of defense response interacted directly or indirectly with the *YrSu* gene to mediate resistance, followed by the signaling molecule SA. When SA level was up-regulated, the H_2_O_2_ scavenging enzyme was activated and SA radicals generated and bound with TaCAT, ROS accumulated and HR appeared. Furthermore, *PR* genes expression increased.

## Author Contributions

XgW, LH, and ZK conceived the study; XgW, XM, XL, XeW, LH, and ZK advised on the experimental design and drafted the manuscript; XgW, YW, PL, YD, MZ, and BH performed the experiments and did the data analysis. YW, YD, PL, MZ, BH, XM, XL, and XeW interpreted data. XgW, LH, and ZK wrote the manuscript and other authors reviewed and revised the manuscript.

## Conflict of Interest Statement

The authors declare that the research was conducted in the absence of any commercial or financial relationships that could be construed as a potential conflict of interest.

## Footnotes

1 http://www.takara.com.cn

2 https://www.thermofisher.com/cn/zh/home/brands/thermo-scientific/molecular-biology/thermo-scientific-molecular-biology-products/fermentas.html?cid=fl-ts-fermentas

## Supplementary Material

**FIGURE S1 | Nulli-tetrasomic analysis by RFLP revealed three homeologs of *TaRar1* in bread wheat genome.**

**FIGURE S2 | Alignment of three homeologs RAR1 proteins from CS.**

**FIGURE S3 | Alignment of DNA sequence of the three *TaRar1* homeologs.** Yellow highlighted region was used for BSMV-VIGS.

**FIGURE S4 | SA concentration** **(A)** and infection type of Suwon11 to CYR31 **(B)**, sporulation **(C)**, and relative fungal DNA to wheat DNA **(D)** after exogenous application of hormones.

**FIGURE S5 | Alignment of DNA sequence of between the *TaRar1* from Suwon11 and the *TaRar1-1* from WGRC7.**
