# Supplementary figures and images for: TaRar1 Is Involved in Wheat Defense against Stripe Rust Pathogen Mediated by YrSu
Source: Front Plant Sci. 2017 Feb 14;8:156. doi: 10.3389/fpls.2017.00156 (PMC5306363; doi:10.3389/fpls.2017.00156)

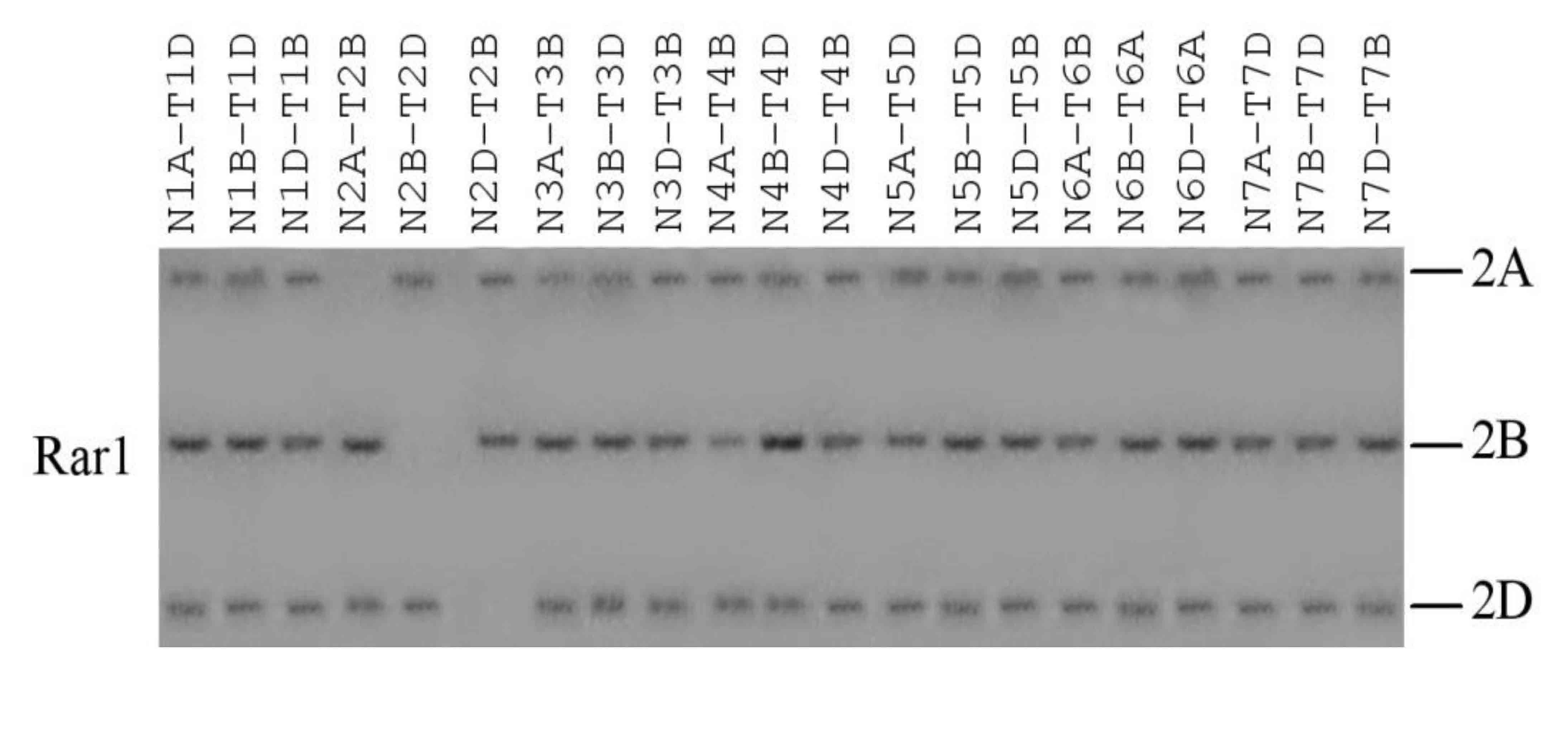

Supplement: Supplementary file 1 [file Image_1.JPEG]

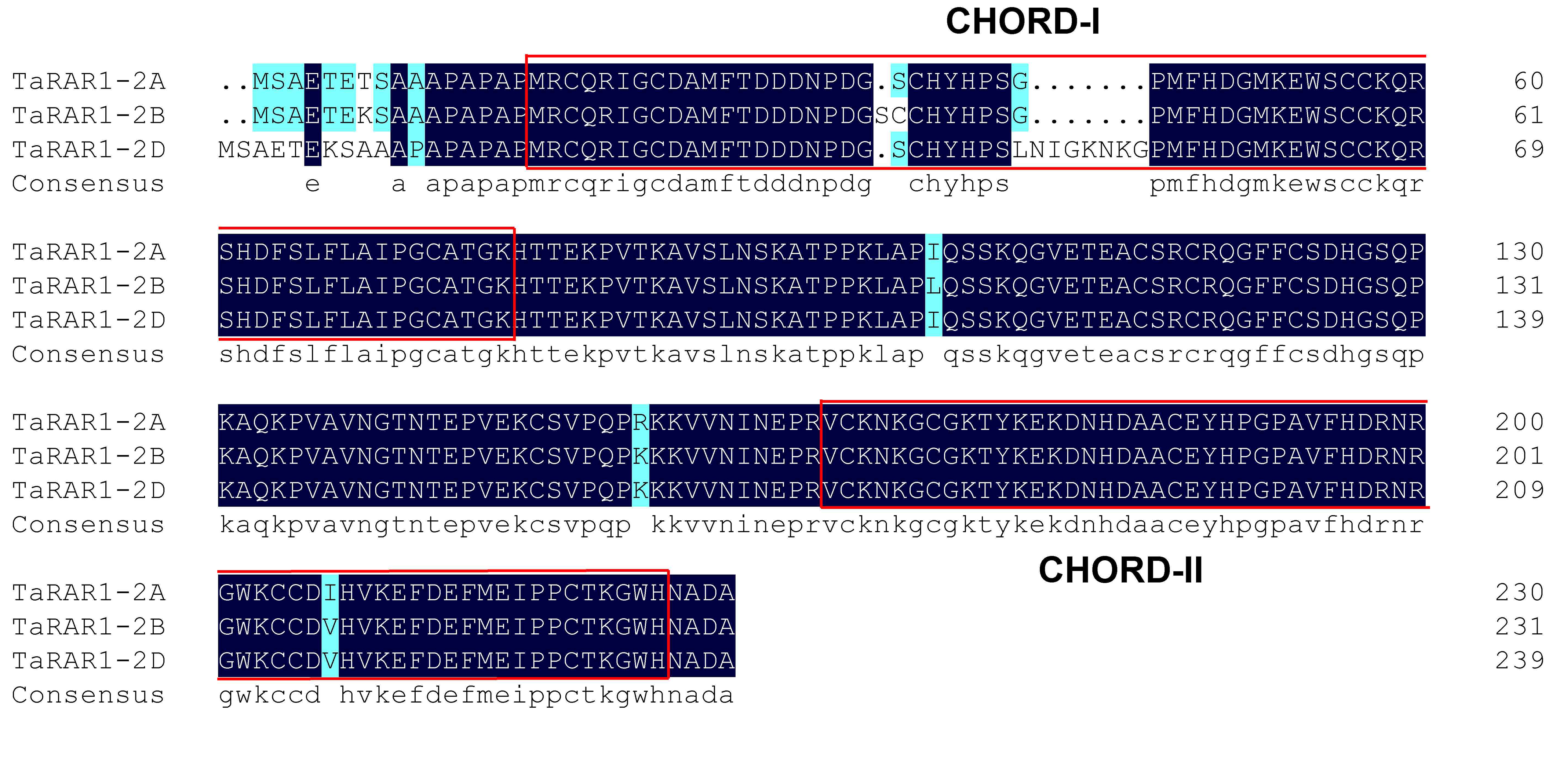

Supplement: Supplementary file 2 [file Image_2.JPEG]

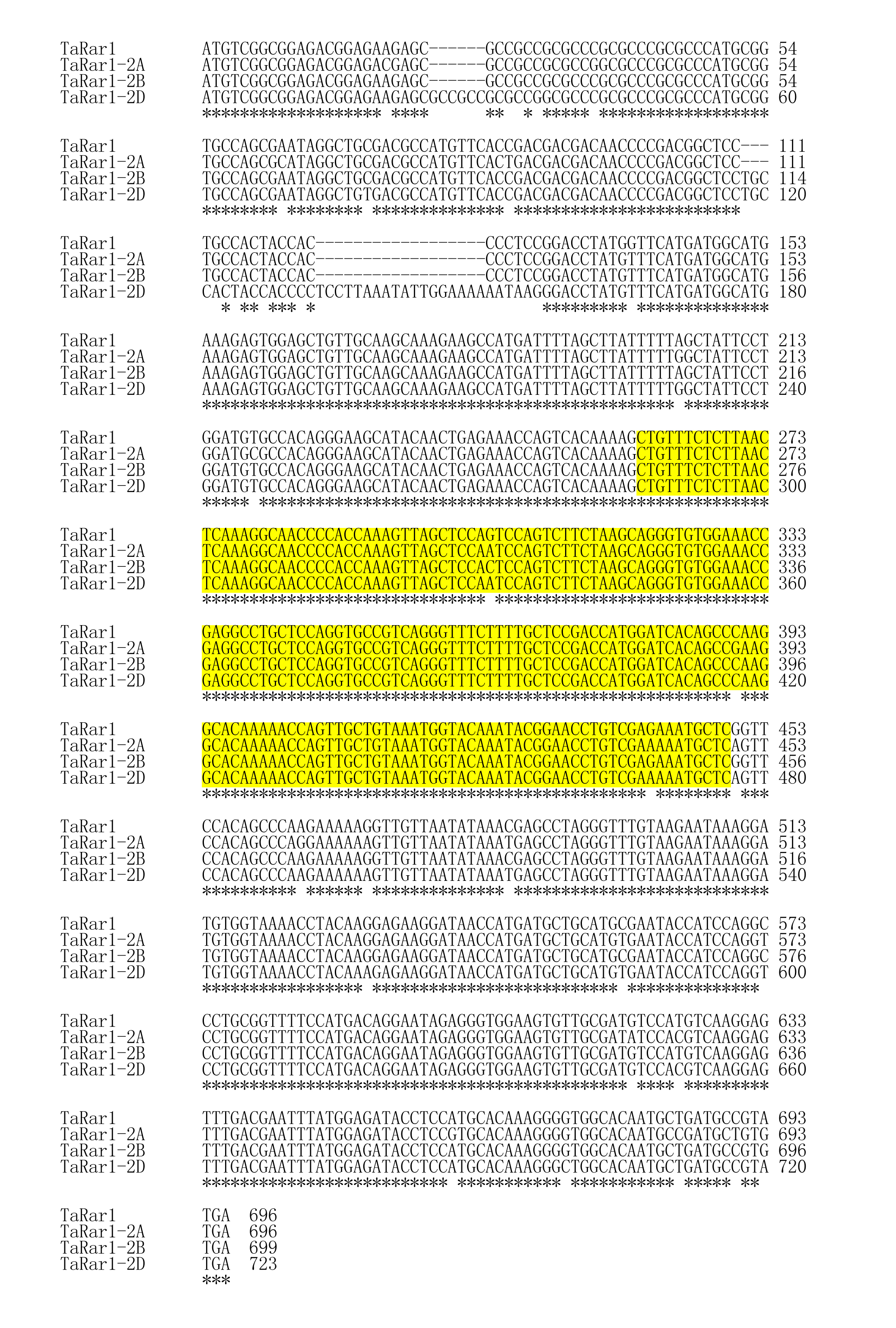

Supplement: Supplementary file 3 [file Image_3.JPEG]

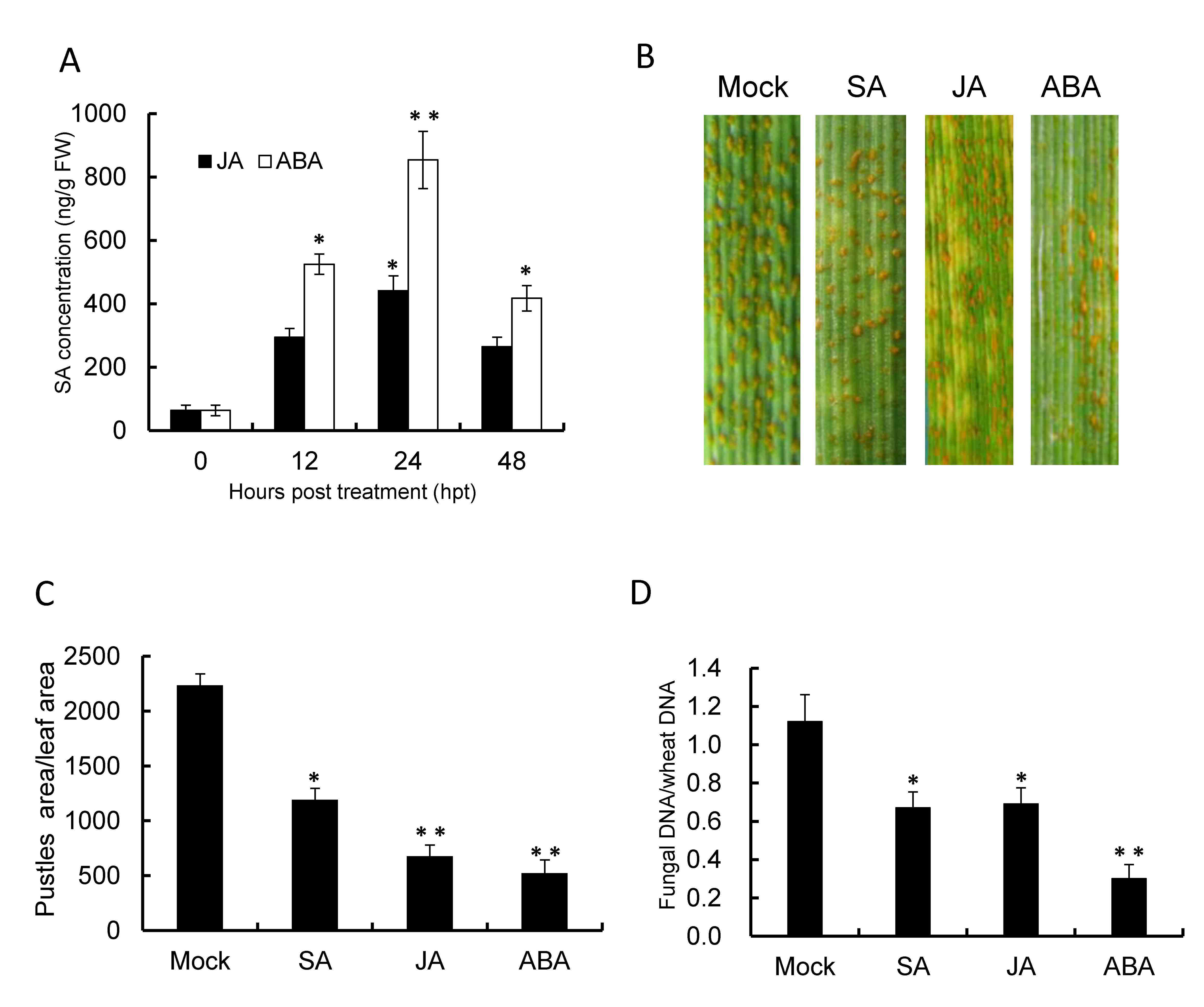

Supplement: Supplementary file 4 [file Image_4.JPEG]

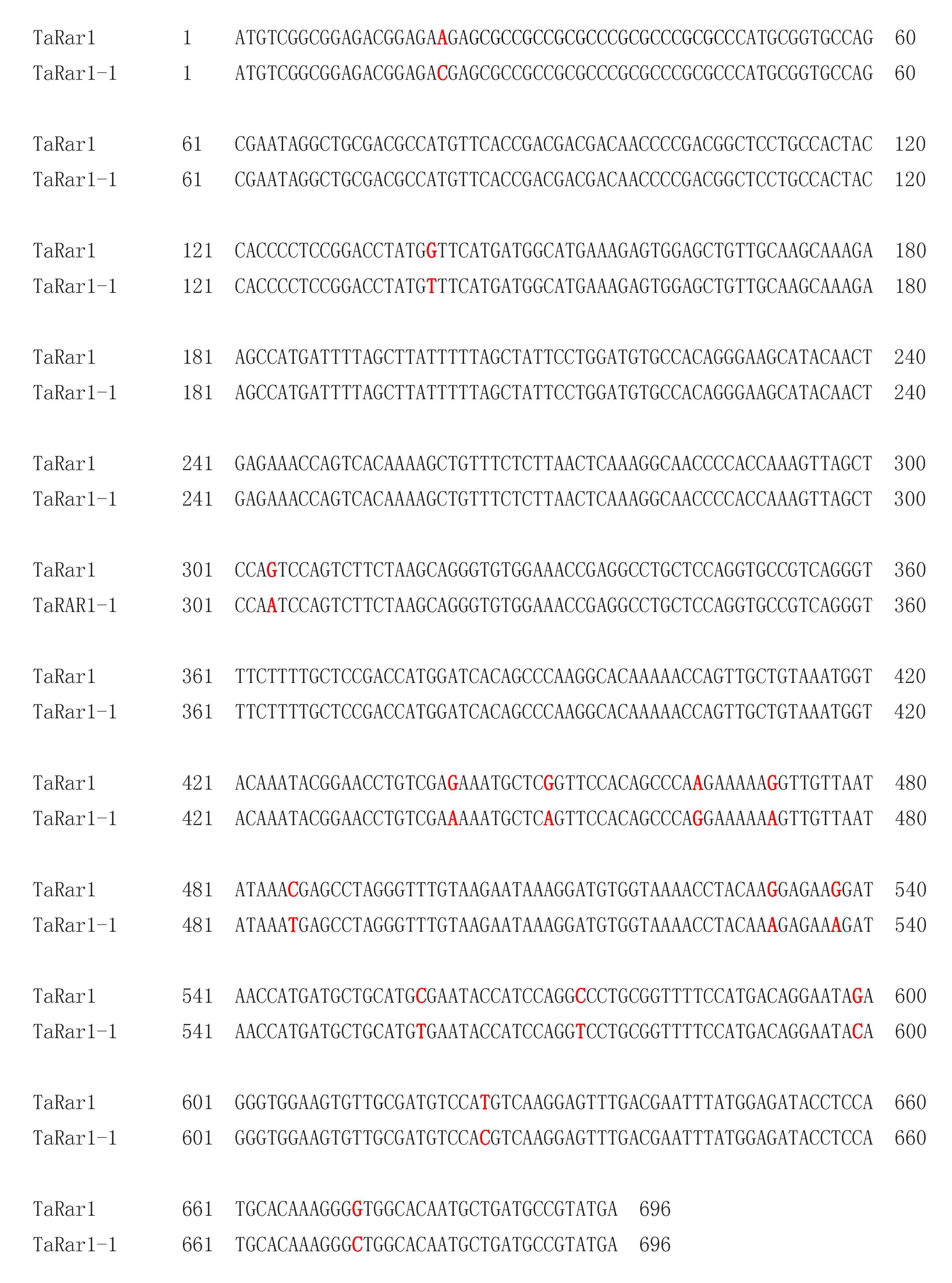

Supplement: Supplementary file 5 [file Image_5.jpg]
